# The Effect of Cognitive Behavioral Therapy‐Based Intervention Program on Psychological Well‐Being and Coping with Stress in Persons With Panic Disorder: Quasi‐Experimental Design With Two‐Site Comparison

**DOI:** 10.1002/brb3.71427

**Published:** 2026-04-17

**Authors:** Tülay Yildirim Üşenmez, Funda Kavak Budak

**Affiliations:** ^1^ Department of Psychiatic and Mental Health Nursing, Atatürk Health Science Faculty Dicle University Diyarbakir Turkey; ^2^ Department of Psychiatric Nursing, Nursing Faculty Inonu University Malatya Turkey

**Keywords:** CBT, coping with stress, panic disorder, psychoeducation, psychological well‐being

## Abstract

**Purpose:**

The current research was made to investigate the effect of an intervention program based on CBT on coping with stress and psychological well‐being in persons with panic disorder.

**Method:**

This research was conducted between May 2021 and July 2022 in a training and research hospital and a university hospital as quasi‐experimental design with two‐site comparison research with a pretest–posttest and control group. The research's participants were 105 persons with panic disorder (54 in the control group and 51 in the experimental group). The Descriptive Features Form, the Psychological Well‐being Scale (PWS), and the coping with stress scale (CSS) were utilized to gather data. The persons in the experimental group performed eight sessions (one session per week) of CBT‐based intervention program in the form of group training, and no training was performed on the persons in the control group.

**Findings:**

The variation between the PWS and the CSS pretest total mean score and the posttest total mean score of the persons in the experimental group was statistically significant (*p* < 0.05).

**Conclusion:**

CBT‐based intervention program can be utilized as an effective psychosocial intervention to enhance coping with stress and promote psychological well‐being in persons with panic disorder.

## Graphical abstract

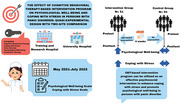

## 1 Introduction

Panic disorder is an ongoing illness with relapses and remissions comprising unexpected panic attacks with physical symptoms such as nausea, dizziness, shortness of breath, and increased heart rate, and cognitive symptoms such as intense anxiety, loss of control, and fear of death (Elkins et al. 2016; Hovland et al. 2015; Wesner et al. 2014). It has been reported that panic disorder has a lifetime prevalence of 1.5%–2% (Das et al. 2020). Persons with panic disorder experience stress because of ongoing thoughts that they will have a panic attack.

The stress experienced with sudden attacks in panic disorder causes persons with this disorder to be unable to fulfill daily life works and decreases their quality of life. Furthermore, it creates severe problems in persons’ social lives and their interpersonal relationships (Barrera and Norton 2009). In the face of these stressful conditions, persons resort to diverse negative and positive ways for coping with stress. These ways may be problem‐focused, be emotion‐focused, involve seeking social support, or focus on escape or avoidance (Kaplan et al. 2012). Katerndahl and Palmer (2000) noticed that persons with panic disorder apply to maladaptive ways for coping with stress. Savoia and Bernik noticed that persons with panic disorder used more ineffective and less adaptive coping ways compared to a control group without mental illness (Savoia and Bernik 2004). Because panic disorder is a multifactorial problem and it can influence persons’ psychological well‐being.

Psychological well‐being is a notion comprising components such as positive personal development, the meaning of life, managing difficulties, and setting effective interpersonal relationships. In other words, psychological well‐being states to one's mental state (Telef 2013). Fava et al. (2001) noticed that persons with panic disorder have lower psychological well‐being than healthy controls. The problems experienced by persons with panic disorder due to their panic attacks make their lives particularly difficult. Evaluating these persons’ coping ways and psychological well‐being can be beneficial in determining the therapeutic effectiveness of treatment (Ağargün et al. 2005).

According to current clinical guidelines, such as those of the National Institute for Health and Care Excellence (NICE, 2020). Cognitive Behavioral Therapy (CBT) is recommended as the first‐line treatment for panic disorder. CBT, which is one such therapy method, is the most preferred therapy method in the panic disorder's treatment (McManus et al. 2008). It is essential to support the positive coping ways used by persons with panic disorder and to change their negative ways using CBT (Ağargün et al. 2005). Wesner et al. (2019) found that CBT increased the coping skills of persons with panic disorder. Researchers have noticed that CBT in the panic disorder's treatment is effective in reducing the intensity of panic disorder symptoms and can prevent relapses (Bruinsma et al. 2016; Feusner et al. 2005; Otto et al. 2016; Poirier‐Bisson et al. 2013). CBT is effective in the panic disorder's treatment. Drug therapy's effectiveness in the panic disorder's treatment has been proven (Pollack 2005). However, it has been determined that some persons refuse to take drugs, experience side effects from drugs, and/or experience relapses during drug treatment, so it is necessary to consider treatment methods other than drug treatment. Therefore, various therapy methods have been considered as methods to complement drug treatment (Nadiga et al. 2003). In this context, psychiatric nurses—who play key roles in education, counseling, case management, and follow‐up—are in a unique position to deliver structured CBT‐based interventions as part of holistic care. Unlike psychotherapists, nurses can incorporate CBT principles into everyday clinical interactions, offering continuous psychological support alongside medical management. Strengthening the role of nurses in implementing CBT‐based interventions may thus contribute to more accessible and sustainable care for individuals with panic disorder.

Although the number of persons with panic disorder increases each day, CBT‐based trainings are still excluded from nursing care plans. This is thought to be due to the lack of CBT training among nurses with postgraduate educations in psychiatric nursing (Y. S. Choi et al. 2017; Demiralp and Oflaz 2007). Psychiatric nurses follow up on persons with mental illness in the area and actively fulfill their main roles, such as education, counseling, case management, rehabilitation, and caregiving. In addition to drug therapy, psychiatric nurses should integrate CBT‐based intervention program into nursing care plans in the panic disorder's treatment due to their educational and counseling roles. However, there is a lack of empirical evidence on the effectiveness of nurse‐delivered CBT components implemented as part of routine psychiatric nursing care. Addressing this gap, the present study aims to evaluate the effect of a nurse‐delivered CBT‐based intervention program on psychological well‐being and coping with stress among individuals with panic disorder. This approach highlights the potential of integrating structured CBT elements into everyday nursing practice, offering an innovative contribution to the existing literature and to the development of evidence‐based nursing care.

### 1.1 Objective

The current research was carried out to evaluate the effect of CBT‐based intervention program on coping with stress and psychological well‐being in persons with panic disorder.

### 1.2 Hypotheses

*H1. CBT‐based intervention program enhances psychological well‐being in persons diagnosed with panic disorder*.

*H2. CBT‐based intervention program enhances the ability to cope with stress in persons diagnosed with panic disorder*.

## 2 Methods

### 2.1 Design and Setting

The research was carried out as quasi‐experimental design with two‐site comparison research with a pretest–posttest and control group. The research was carried through from May 2021 to July 2022 in the psychiatry outpatient clinics of a training and research hospital (TRH) and a university hospital (UH).

### 2.2 Population and Sample

The research universe comprised 550 persons diagnosed with panic disorder who were registered with the TRH's and UH's psychiatry outpatient clinics. Utilizing G*Power, the convenient sample size was determined to be 100 persons (50 in the control group and 50 in the experimental group) with panic disorder, a confidence interval of 0.95, an effect size of 0.9, with an error of 0.05, and a universe representation power of 0.95 (Wesner et al. 2014). A lottery was drawn to determine which hospital the control and experimental groups would come from. It was decided that the persons registered with the TRH would form the experimental group and that the persons registered with the UH would form the control group.

**FIGURE 1:**
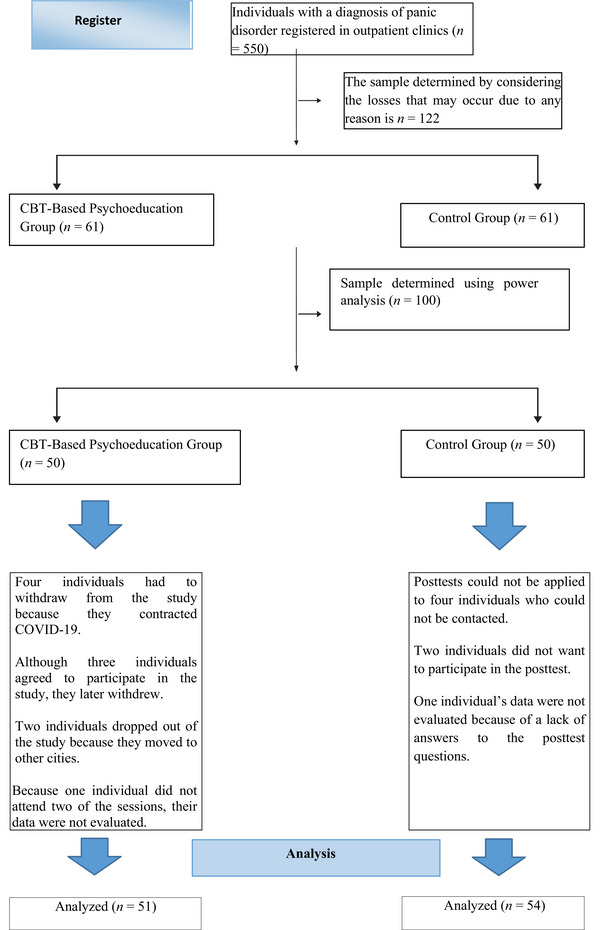
CONSORT flow diagram.

This study was designed as quasi‐experimental design with two‐site comparison research with a pretest–posttest and control group. Participants were recruited from two comparable psychiatric hospitals that served similar patient populations and operated under similar treatment protocols. To prevent contamination between groups, all eligible individuals from TRHs were allocated to the experimental group, while all eligible individuals from UHs were allocated to the control group. A total of 122 individuals with panic disorder were initially included in the study (61 in the experimental group and 61 in the control group), considering possible dropouts. Ultimately, 105 participants (51 in the experimental group and 54 in the control group) completed the study. The CONSORT flow diagram illustrating participant progress through each stage of the trial is presented in Figure 1.

### 2.3 Inclusion Criteria

Inclusion criteria are as follows:

- being diagnosed with panic disorder (according to DSM‐5);
- Being 18 years or older;
- Being able to be literate.

### 2.4 Exclusion Criteria

Exclusion criteria are as follows:

- Being diagnosed with dementia, Alzheimer's, mental retardation, head trauma, etc. registered in the individual's file has situations;
- Having language or any disability that prevents communication;
- Having attended CBT‐based training at least 6 months ago;
- Having a psychiatric diagnosis (according to the DSM‐5) other than panic disorder (Individuals with any additional psychiatric diagnoses were excluded to ensure that the intervention's effects could be attributed specifically to panic disorder symptoms. This approach was intended to minimize confounding factors that might arise from comorbid conditions requiring different therapeutic approaches or medications).

### 2.5 Measures

#### 2.5.1 Descriptive Features Form (DFF)

The researcher created the DCF according to the literature (Wesner et al. 2019). The form consisted of eight questions (gender, employment status, age, marital status, education level, family history of mental illness, time of first diagnosis, and duration of treatment).

#### 2.5.2 Psychological Well‐Being Scale (PWS)

It was improved by Diener et al. (2010). Telef carried out research on the scale to confirm its reliability and validity within a Turkish context (Cronbach's *α* 0.80) (Telef 2013). The PWS consists of eight items and a single dimension measured on a seven‐point Likert‐type scale. The total scores range from 8 to 56. High scores showed a high level of psychological well‐being. For the purpose of the current research, Cronbach's α was calculated as 0.95.

#### 2.5.3 Coping With Stress Scale (CSS)

The original CSS scale (based on the mental stress model) improved by Folkman and Lazarus. The reliability and validity research within a Turkish context was verified by Türküm (Cronbach's α 0.78) (Türküm 2002). The scale was 5‐point Likert type and occured twenty‐three items and three dimensions. Three items (10, 17, and 20) were scored by reversing. The total score ranges from 23 to 115; the total score for the problem‐focused dimension ranges from 1 to 40, the total score for seeking social support dimension ranges from 1 to 35; and the total score for the escape‐avoidance dimension ranges from 1 to 40. The scores acquired from the dimensions gave knowledge about persons' methods for coping with stress. For the purpose of the current research, Cronbach's α was calculated as 0.69.

### 2.6 Data Collection

Data were gathered via face‐to‐face meeting with persons with panic disorder in the training hall once a week. Due to the pandemic, social distancing was observed during this training, and both the researcher and the interviewees wore masks. The training hall was also properly ventilated, and disinfectant was made available at the entrance. The researcher distributed data collection forms to persons, and they filled them out. It took each individual approximately 15–20 min to fill out these forms. For the research's experimental group, the application of the pre‐test forms (DFF, PWS, and CSS) was in May 2021, and the application of the post‐test forms (PWS and CSS) took place in October 2021, 2 weeks after the training was completed. The research's control group completed the pre‐test form applications (DFF, PWS, and CSS) in May 2021 and the post‐test forms application (PWS and CSS) in November 2021.

### 2.7 Nursing Intervention

The CBT‐based psychoeducation training booklet supported interactive training was conducted for the experimental group. To be able to perform CBT‐based intervention program, the researcher had to get certification via a training program refered to as “Applied CBT Training.”

The experimental group was seperated eight groups, each containing five to eight participants. The researcher ensured face‐to‐face and group training once a week for 8 weeks. Each seance was 50–60 min on average. Additionally, make‐up sessions were carried out for persons who could not join the original sessions. Education question‐answer, case studies lectures, role‐play, etc., were carried out interactively using methods.

No training was performed on the control group persons. During this time, persons in the experimental and control groups sustained their medical treatments.

### 2.8 CBT‐Based Intervention Program Sessions Content

**FIGURE 2:**
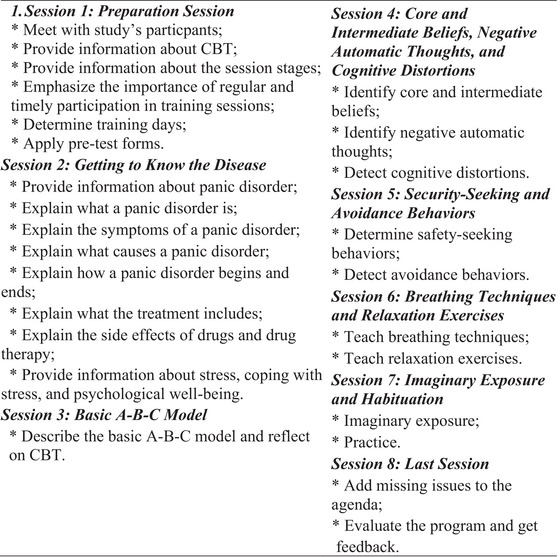
CBT‐based intervention program plan.

The researcher prepared the CBT‐based intervention program's content in line with the literature (Wesner et al. 2014). The research's CBT‐based intervention program's content is shown in Figure 2. The thematic focus of each session is as follows:

**Table:** 

Session	Theme/focus	Key techniques/activities
1	Introduction	Preparation intervention
2	Psychoeducation about panic disorder	Overview of symptoms, disease course, and treatment rationale
3	Cognitive restructuring I	Identifying automatic thoughts and cognitive distortions
4	Cognitive restructuring II	Challenging negative thoughts, cognitive rehearsal exercises
5	Stress management & relaxation	Breathing techniques, progressive muscle relaxation
6	Imaginative exposure	Gradual exposure to panic‐inducing situations in imagination
7	Coping strategies & relapse prevention	Problem‐solving, behavioral planning, and coping skills reinforcement
8	Closing	Evaluate the Intervention

### 2.9 Analysis

SPSS 25.0 version was utilized for analyzing data. A *p*‐value of ˂ 0.05 was taken notice of as significant for the research. The internal consistency analysis of the scales was examined by Cronbach's α coefficient. Kolmogrov–Smirnov and Shapiro–Wilks tests were utilized along with a histogram, *Q*–*Q* plot, *P*–*P* plot, and an evaluation of skewness and kurtosis to evaluate the conformity with normal distribution. The results of the analysis indicated that the data had a normal distribution. The experimental and control groups’ percentage of distribution, arithmetic mean, chi‐square test, standard deviation, min‐max values, PWS, and CSS mean scores were all utilized to evaluate the descriptive features of the persons in these groups. The dependent samples *t*‐test was used for within‐group, and independent samples *t*‐test was used for between‐group.

### 2.10 Ethical Declaration

Firstly, confirmation was acquired from the ethics committee of a university (Number: 2021/1950), and official permissions were acquired from the hospitals. The purpose of the research was clarified to the person with panic disorder, and they were notified that their data would be protected confidentially and that they could leave the study at any time. Additionally, written consent was acquired from the persons utilizing an informed voluntary consent form. After the post‐test data were gathered and the experimental group was ensured of CBT‐based psychoeducation, CBT‐based psychoeducation booklets were given to the persons from the control group upon request.

## 3 Results

Taking account of the descriptive features of the persons in the experimental and control groups, 37.3% of the persons in the experimental group were 29–39 years old, 64.7% were female, 64.7% were married, 37.3% were high school graduates, and 51.0% were working. Moreover, 62.7% had incomes equal to their expenditures, 74.5% had no family history of mental illness, 45.1% had a 0–1 year diagnosis of the disease, and 49.0% had treatment periods of 0–1 year. Of the persons in the control group, 40.7% were 29–39 years old, 59.3% were female, 61.1% were married, 31.5% were high school graduates, 59.3% were working, 66.7% had incomes equal to their expenditures, and 70.4% had no family history of mental illness. Moreover, 38.9% of them were diagnosed with the disease in 2–5 years, and 42.6% of them had treatment periods of 0–1 year. The experimental and control groups were homogeneous in terms of control variables (*p* > 0.05; Table 1).

**TABLE 1:** Comparison of the control variables of experimental and control groups (*N* = 105).

Experimental group (*N* = 51)			Control group (*N* = 54)		Test and significance^a^
Descriptive characteristics	S	%	S	%	
**Age groups**					
18–28 years	14	27.5	13	24.1	
29–39 years	19	37.3	22	40.7	*x* ^2^ = 1.099
40–50 years	15	29.4	15	27.8	*p* = 0.982
51 and above	3	5.9	4	7.4	
**Gender**					
Female	33	64.7	32	59.3	*x* ^2^ = 0.139
Male	18	35.3	22	40.7	*p* = 0.709
**Marital status**					
Married	33	64.7	33	61.1	*x* ^2^ = 0.032
Single	18	35.3	21	38.9	*p* = 0.858
**Education level**					
Primary school	5	9.8	7	13.0	
Secondary school	14	27.5	14	25.9	*x* ^2^ = 0.720
High school	19	37.3	17	31.5	*p* = 0.888
University	13	25.5	16	29.6	
**Working status**					
Employed	26	51.0	32	59.3	*x* ^2^ = 0.431
Unemployed	25	49.0	22	40.7	*p* = 0.512
**Perception of income level**					
Income less than expenses	19	37.3	18	33.3	*x* ^2^ = 0.047
Income equal to the expenses	32	62.7	36	66.7	*p* = 0.829
**Family history of mental illness**					
Yes	13	25.5	16	29.6	*x* ^2^ = 0.065
No	38	74.5	38	70.4	*p* = 0.798
**Time of first diagnosis**					
0–1 years	23	45.1	17	31.5	
2–5 years	18	35.3	21	38.9	*x* ^2^ = 4.948
6–10 years	10	19.6	12	22.2	*p* = 0.170
11–15 years	0	0.0	4	7.4	
**Duration of treatment**					
0–1 years	25	49.0	23	42.6	
2–5 years	17	33.4	17	31.9	*x* ^2^ = 2.844
6–10 years	9	17.6	11	20.4	*p* = 0.464
11–15 years	0	0.0	3	5.1	

^a^Chi‐square test.

The difference between the experimental and control groups’ pretest total mean scores on the PWS and the CSS was not statistically significant (*p* > 0.05; Table 2).

**TABLE 2:** Comparison of the pre‐test PWB and CSS subscales and total mean scores of the experimental and control groups (*N* = 105).

Pre‐test			
	Experimental group (*N* = 51)	Control group (*N* = 54)	Test and significance^a^
	(X ± SS)	(X ± SS)	
**PWB** **CSS** Seeking social support Problem‐focused Escape‐avoidance Total	30.60 ± 7.62 29.82 ± 4.22 23.43 ± 5.75 30.78 ± 5.45 84.03 ± 5.98	30.68 ± 8.28 29.98 ± 4.19 23.03 ± 5.48 30.68 ± 5.56 83.70 ± 6.39	*t* = −0.050 *p* = 0.960 *t =* −0.192 *p* = 0.848 *t =* 0.360 *p* = 0.720 *t =* 0.092 *p* = 0.927 *t =* 0.277 *p* = 0.782

^a^Independent sample *t* test.

The difference between the groups’ posttest total mean scores was statistically significant (*p* ˂ 0.05, Table 3).

**TABLE 3:** Comparison of the post‐test PWB and CSS subscales and total mean scores of the experimental and control groups (*N* = 105).

POST‐TEST			
	Experimental group (*N* = 51)	Control group (*N* = 54)	Test and significance^a^
	(X ± SS)	(X ± SS)	
**PWB** **CSS** Seeking social support Problem‐focused Escape‐avoidance Total	41.37 ± 4.06 29.27 ± 4.09 31.92 ± 2.24 25.52 ± 3.98 86.72 ± 6.84	31.46 ± 8.35 30.04 ± 4.09 23.52 ± 5.46 30.01 ± 5.25 83.57 ± 5.96	*t* = 7.655 ***p* = 0.000** *t =* −1.069 *p* = 0.288 *t =* 10.069 ***p* = 0.000** *t =* −5.780 ***p* = 0.000** *t =* 1.719 ***p* = 0.000**

^a^Independent sample *t* test.

*p*<0.05

The difference between the groups’ pretest and posttest total mean PWS scores was statistically significant (*p* < 0.05; Table 4). The difference between the experimental group's pretest and posttest total mean CSS scores was statistically significant (*p* < 0.05, Table 4), whereas the difference between the control group's pretest and posttest total mean CSS scores was not statistically significant (*p* > 0.05, Table 4).

**TABLE 4:** Comparison of the pretest–posttest PWS and CSS subscales and total mean scores of the experimental and control groups (*N* = 105).

Groups	Pre‐test (X ± SS)	Post‐test (X ± SS)	Test and significance^a^
			
**PWS**			
Experimental group (*N* = 51)	30.60 ± 7.62	41.37 ± 4.06	*t =* −11.629 ***p* = 0.000**
Control group (*N* = 54)	30.68 ± 8.28	31.46 ± 8.35	*t =* −2.321 ***p* = 0.024**
**CSS**			
Experimental group (*N* = 51)	29.82 ± 4.22	29.27 ± 4.09	*t =* 2.524 ***p* = 0.015**
Seeking social support	23.43 ± 5.75	31.92 ± 2.24	*t =* −10.972 ***p* = 0.000**
Problem‐focused	30.78 ± 5.45	25.52 ± 3.98	*t =* 10.660 ***p* = 0.000**
Escape‐avoidance Total	84.03 ± 5.98	86.72 ± 6.84	*t* = −4.628 ***p* = 0.000**
Control group (*N* = 54)			
Seeking social support	29.98 ± 4.19	30.04 ± 4.09	*t =* −1.827 *p* = 0.073
Problem‐focused	23.03 ± 5.48	23.52 ± 5.46	*t* = −2.930 *p* = 0.065
Escape‐avoidance	30.68 ± 5.56	30.01 ± 5.25	*t* = −0.880 *p* = 0.383
Total	83.70 ± 6.39	83.57 ± 5.96	*t* = −4.021 *p* = 0.054

^a^Dependent sample *t* test.

*p*<0.05

## 4 Discussion

In the current section, the findings of this research, which was carried out to evaluate the effect of CBT‐based intervention program on psychological well‐being and coping with stress in persons with panic disorder, are examined according to the literature.

### 4.1 Findings Related to Psychological Well‐Being

The difference between the total mean PWS scores of the persons in the control and experimental groups before CBT‐based intervention program was statistically insignificant. Fava et al. (2001) reported that persons with panic disorder have lower psychological well‐being than healthy controls. Very few studies have examined the psychological well‐being levels of persons with panic disorder. For this reason, the findings were discussed with studies examining psychological well‐being levels in persons with various mental illnesses. Iani et al. (2019) declared that the symptoms of generalized anxiety disorder were negatively associated with psychological well‐being. Kim et al. (2018) stated that psychological well‐being levels were low among psychiatric patients. The differences among the studies may be because the studies were focused on different psychiatric diagnoses.

After CBT‐based intervention program, the difference between the experimental and control groups in terms of total mean PWS scores was statistically significant. Studies viewing the effect of CBT‐based intervention program on psychological well‐being in persons with panic disorder could not be found.

The difference between the intragroup pretest and posttest total mean PWS scores of the persons in the experimental and control groups was statistically significant. The increase in the posttest PWS scores of the persons in the control group may be because those persons could easily access treatment opportunities, continue their medical treatment, and receive social support from their environment. Persons with panic disorder experience distress because of the panic attacks they experience. This not only makes them unable to fulfill daily life activities and decreases their quality of life but also creates serious problems in their interpersonal relationships and their social and business lives. According to Ryff's definition, persons cannot focus on psychological well‐being dimensions and thus are unable to establish positive relationships, fail to realize personal development, lose environmental dominance, lose their life purpose, struggle with being autonomous, and see their weaknesses in the face of negative situations (Fava et al. 2001; Geçgin and Sahranc 2017). Techniques specific to CBT include psychoeducation and mindfulness, cognitive restructuring, relaxation exercises, and breathing techniques. persons can be taught to recognize distorted thoughts and schemas—such as thoughts that the normal bodily sensations they experience during panic attacks are fatal—so they can change these thoughts, which is effective in reducing anxiety and avoidance behavior. Thus, persons can start to establish more positive relationships with their environment, accept themselves, and discover their life purpose. As a result of their CBT‐based psychoeducation, the experimental group's increase in total mean PWS scores proved the research hypothesis H1.

### 4.2 Discussing Findings Related to Coping With Stress

The difference between the experimental and control groups in terms of pretest total mean CSS scores before CBT‐based intervention program was statistically insignificant. It has been reported that persons with panic disorder frequently use negative ways—such as avoidance, self‐blame, and wishful thinking—to cope with stress (Hino et al. 2002; Vitaliano et al. 1987; Yamada et al. 2004). Wishful thinking may cause persons to be unable to adapt to panic disorder (Hino et al. 1998, 1999). Another coping way used by persons with panic disorder is seeking social support. It has been stated that support‐seeking ways, which include asking for help from others, can provide psychological support to persons who resist adapting to panic disorder and thus enable them to cope positively (Vollrath and Angst 1993). In 1‐year follow‐up research conducted with persons with panic disorder, Hino et al. declared that persons with panic disorder scored higher in coping ways such as escape‐and‐avoidance, seeking social support, and emotion‐focused coping ways than healthy persons in the first evaluation. After 1 year, there was no change in these scores. This shows that ways for coping with stress associated with panic disorder include seeking social support, emotion‐focused ways, and escape and avoidance (Hino et al. 2002). Vitaliano et al. (1987) declared that persons with panic disorder utilize less problem‐focused ways and more wishful thinking ways than healthy persons. Vollrant et al. stated that persons with panic disorder apply to seeking social support, escape‐and‐avoidance ways, and wishful thinking ways more than healthy persons and persons with other anxiety disorders (Vollrath and Angst 1993). Cox et al. (1992) observed that persons with panic disorder frequently utilize escape and avoidance to coping with stress. The ways that persons with panic disorder use to cope with stressful situations are ineffective and inappropriate (Savoia and Bernik 2004).

After CBT‐based intervention program, the change in the experimental group's total mean CSS score was statistically significant. Using maladaptive ways to cope with stressful events may trigger relapses in symptoms’ appearance and persistence (Manfro et al. 1996; Moitra et al. 2011). According to hypotheses regarding CBT's role in the treatment of panic disorder, psychosocial interventions—especially cognitive methods—can be utilized to help persons with panic disorder develop effective coping ways (Hyun et al. 2010; Padesky and Mooney 2012; Songprakun and McCann 2012). Persons with panic disorder tend to cope with their stress using ways such as escape‐and‐avoidance ways, wishful thinking, self‐blame, and ignoring their problems (Ramage‐Morin 2004). In their research on persons with panic disorder, Wesner et al. (2019) provided 12 sessions of cognitive behavioral group therapy to an experimental group and a control group. The experimental group also underwent four sessions of coping ways and resilience training. Both groups demonstrated significant decreases in symptom severity and maladaptive ways for coping with stress (Wesner et al. 2019). The coping skills taught in CBT are aimed at changing bodily symptoms and catastrophic assessments. Persons are encouraged to practice bodily skills (e.g., breathing and relaxation exercises) to control physiological symptoms and cognitive skills that control negative thoughts (e.g., recognizing negative automatic thoughts) by exposing them to bodily sensations and fear (Meuret et al. 2012; Norton & Price 2007; Westen and Morrison 2001). The CBT performed in the current research included mediators like informational psychoeducation on panic disorder, somatic management skills including progressive muscle relaxation training and diaphragmatic breathing, adjusting cognitive distortions, and interoceptive and in vivo exposures. These mediators are a treatment method that works by finding the wrong thoughts via a systematic approach and changes them into reasonable thoughts (Beck and Dozois 2011). In detail, persons with panic disorder study how to control tension and anxiety by tightening and relaxing the muscles through diaphragmatic breathing and progressive muscle relaxation training. Persons with panic disorder breathe faster than the norm, and panic attacks can be decreased by breathing slower (Y. H. Choi et al. 2003).

The difference between the experimental group's pretest and posttest total mean CSS scores was statistically significant. The ways for coping with stress vary from person to person. Two types of ways are generally used in coping with stress: problem‐focused ways and emotion‐focused ways. In problem‐focused ways, a person takes responsibility by seeking a solution to the problem while trying to eliminate or reduce the stressor's effect and takes measures to avoid encountering the stressor again. Such ways include activities aimed at directly changing the factors that create stressful situations, such as concentrating on solutions. These activities include conscious processes and active behavioral steps, such as identifying the problem, producing alternative solutions, evaluating these alternative solutions’ positive and negative aspects, and applying one of the solutions (Yerlikaya 2009). In emotion‐focused coping, persons demonstrate behaviors such as delaying their emotions, crying, eating and drinking, denial, escape and avoidance, seeking social support, avoiding the problem, and avoiding thinking about the problem (Türküm 2002). Before the CBT‐based psychoeducation, persons in both groups coped with their stress using emotion‐focused ways, especially escape and avoidance. CBT‐based intervention program is aimed at teaching persons how thought processes take place (according to the ABC [adversity–beliefs–consequences] model), how to identify cognitive distortions and automatic thoughts with the Socratic questioning method, and how to use methods to produce alternatives. persons are supported in developing their problem‐solving skills and using problem‐focused ways to cope with stress. The increase in the experimental group's total mean CSS score resulting from the CBT‐based intervention program approved the research hypothesis H2.

Our findings are consistent with previous studies demonstrating the effectiveness of CBT‐based interventions in improving coping skills and psychological well‐being among individuals with panic disorder. For instance, Westen et al. (2019) reported that CBT increased coping skills, while Bruinsma et al. (2016) and Otto et al. (2016) found reductions in panic symptom severity and relapse prevention. Unlike most previous studies, which focused on therapist‐delivered CBT, our study demonstrates that nurse‐delivered CBT‐based intervention programs can also yield significant benefits. This suggests that incorporating trained psychiatric nurses into routine care may enhance accessibility and continuity of psychological interventions for individuals with panic disorder.

Despite the promising findings of this study, several limitations should be considered. First, randomization occurred at the cluster (hospital) level rather than at the individual level, which carries a potential risk of systematic differences between clusters. Although the hospitals were similar in demographic and organizational characteristics, site‐level effects could not be entirely excluded. Due to the small number of clusters, formal adjustment for intra‐cluster correlation was not feasible. Second, all psychiatric comorbidities were excluded. While this strengthened internal validity by isolating the effects of the intervention, it may have reduced ecological validity, as comorbid anxiety and mood disorders are common in individuals with panic disorder. Future studies should consider including participants with mild to moderate comorbidities to better reflect real‐world clinical populations and enhance generalizability. Third, the study was not preregistered. Although this does not affect the ethical integrity of the research, preregistration is increasingly recognized as a way to enhance methodological transparency and prevent selective reporting. Future research should therefore consider preregistration to strengthen reproducibility and credibility. Finally, the absence of a formal time × group interaction analysis may limit the precision of the conclusions. Future studies with larger, multi‐site samples should employ repeated‐measures designs or linear mixed models to better account for time and group effects simultaneously. These limitations should be considered when interpreting the findings, but they do not undermine the observed benefits of the nurse‐delivered CBT‐based intervention in improving coping and psychological well‐being among individuals with panic disorder.

A notable observation in this study was the high degree of baseline equivalence achieved between the intervention and control groups, despite the limited number of randomized clusters (*n* = 2). This statistical stability can be attributed to two main factors. First, the stringent exclusion criteria acted as a rigorous filter, removing the “noise” typically introduced by psychiatric comorbidities such as clinical depression or personality disorders. By isolating a homogeneous population of individuals with primary panic disorder, the inherent variance between the two groups was naturally minimized. Second, the institutional symmetry between the selected hospitals played a crucial role. Both sites operate under identical national clinical guidelines and serve populations with similar socioeconomic and cultural backgrounds. These factors collectively functioned as a structural control mechanism, ensuring that the participants entered the study with comparable psychological profiles and baseline symptoms, thereby strengthening the internal validity of the intervention's effects.

## 5 Limitations

This study has several methodological limitations that warrant careful interpretation. His study has several limitations that should be acknowledged. First, the intervention was conducted using a quasi‐experimental design with two‐site allocation rather than individual randomization. This approach may lead to a potential confounding effect between the treatment and the specific institutional characteristics of the hospitals (TRH vs. UH). Second, the sample size calculation was performed at the individual level and did not account for the intraclass correlation coefficient or the design effect, which is typically required for cluster‐based designs. Therefore, the statistical power may be lower than initially estimated, and the findings should be interpreted as preliminary evidence from a pilot investigation. Future research involving a larger number of clusters and multi‐center randomization is needed to confirm the generalizability of these results. Although the two hospitals were highly similar in socio‐demographic, cultural, and organizational characteristics and followed identical national treatment protocols, site‐level effects cannot be entirely excluded. Due to this constraint, formal adjustment for intra‐cluster correlation (ICC) was not feasible, and the study may be viewed as a pilot multi‐center comparison or a prospective feasibility study rather than a definitive large‐scale RCT. Although the hospitals were similar in socio‐demographic/economic/cultural and organizational characteristics and followed identical national treatment protocols, site‐level effects could not be entirely excluded. Due to the small number of clusters, formal adjustment for intra‐cluster correlation was not feasible. Second, the exclusion of all psychiatric comorbidities, while strengthening internal validity by isolating the effects of the intervention, may have reduced ecological validity. In real‐world clinical settings, comorbid anxiety and mood disorders are highly prevalent among individuals with panic disorder. Future studies should consider including participants with mild to moderate comorbid conditions to better reflect routine clinical populations and enhance generalizability. Third, the study was not preregistered. Although this does not affect the ethical integrity of the research, preregistration is recognized as an important step to enhance methodological transparency and prevent selective reporting. Future studies should therefore consider preregistration to strengthen reproducibility and credibility. Finally, the absence of a formal time × group interaction analysis may limit the precision of the conclusions. Future studies with larger, multi‐site samples should employ repeated‐measures designs or linear mixed models to better account for time and group effects simultaneously. Another limitation is related to the statistical approach. The current analysis relied on individual‐level t‐tests, which do not formally account for the time × group interaction or the intra‐cluster correlation inherent in a two‐site design. This may lead to an underestimation of standard errors and potential inflation of significance levels. Therefore, the results should be viewed as preliminary evidence of the intervention's potential, and future studies with more clusters are required to apply more sophisticated mixed‐effects modeling.

## 6 Conclusion and Recommendations

Before the CBT‐based intervention program, persons in both groups coped with their stress using emotion‐focused ways, like as escape and avoidance. Based on the research's results, CBT‐based intervention program may be utilized as an effective psychosocial intervention to enhance psychological well‐being and the ability to cope with stress in persons with panic disorder. In line with these results, psychoeducation based on CBT‐based interventions can be combined with the nursing care plans for persons with panic disorder—in addition to their medical treatments—to enhance their psychological well‐being and coping skills. Additionally, it may be helpful to conduct similar studies using larger samples.

## Author Contributions

**Tülay Yildirim Üşenmez**: Conceptualization, Investigation, Writing – original draft, Methodology, Writing – review and editing, Formal analysis, Data curation, Resources, **Funda Kavak budak**: writing – review and editing, methodology, supervision, formal analysis.

## Funding

The authors have nothing to report.

## Ethics Statement

Before starting the study, approval was obtained from the health sciences non‐invasive clinical trial ethics committee of Inonu University (Approval No: 2021/1950), and legal permissions were obtained from the hospitals where the study was conducted. The individuals with panic disorder were informed of the purpose of the study and assured that their information would be kept confidential, and they could withdraw from the study at any time. They were then asked to provide written and verbal consent. Their written consents were obtained through Informed Voluntary Consent Form. All procedures were carried out in accordance with the ethical rules and the principles of the Declaration of Helsinki. Clinical trial number: not applicable.

## Conflicts of Interest

The authors declare no conflicts of interest.

## Data Availability

All data supporting the findings of this study are available within the paper.
